# Spread of Infectious Disease.—II

**Published:** 1904-09-03

**Authors:** J. E. R. Stephens

**Affiliations:** Author of "Digest of Public Health, Case-Law," etc.


### SPREAD OF INFECTIOUS DISEASE.—II.

By J. E. R. Stephens, Barrister-at-Law, Author of "Digest of Public Health, Case-Law," etc.

(*Concluded from page* 160.)

### Compensation for Loss of Employment.

In considering the legal aspects of this question, the first thing to be remembered is the precise scope of Sect. 308 of the Public Health Act, 1875, which gives power to a local authority to award compensation in certain cases to persons damaged by the enforcement of the provisions of the statute. That section enacts that where any person sustains any damage by reason of the exercise of any of the powers of this Act in relation to any matter as to which he is not himself in default, full compensation shall be made to such person by the local authority exercising such powers. Therefore, in order to prove his right to full compensation, a person must show: (1) that he has sustained damage, *i e.*, pecuniary loss; (2) that the damage has been sustained by him by reason of the exercise of some of the powers conferred upon the sanitary authority by the Public Health Acts; and (3) that such damage was sustained by him in relation to a matter as to which he was not himself in default.

No doubt loss of wages would be "damage" within the meaning of the section. The real difficulty in the great majority of cases lies in the proof that the loss of wages was sustained by reason of the exercise by the sanitary authority of some of the powers conferred upon them by the Acts relating to public health. In the case of *Roberts* v. *Falmouth Sanitary Authority* (52 J.P. 741) the urban sanitary authority, owing to an epidemic of measles, ordered a board school to be closed for a fortnight, whereby the schoolmaster lost his fees, amounting to 30s. a week. He claimed compensation under Sect. 308 of the Public Health Act, 1875, but it was held that he had no valid claim, the power to close the school being given by the Education Code, 1886, Sect. 98, and not by the Public Health Acts. There is, however, no direct power given by any statute to a local authority to prevent a man going to his business when a member of his family is suffering from a dangerous infectious disease. Of course, if the man was himself suffering from a dangerous infectious disease, he might be prosecuted for wilfully exposing himself without proper precautions against spreading the disorder in any street, etc, as enacted by Sect. 126 of the Public Health Act, 1875. Or if he exposed the suffering member of his family in a similar manner, he might be prosecuted as the person in charge of such member. But being himself in health, and leaving the sick member of his family at home, the only possible offence for which he could be prosecuted if he went to his work would be for exposing without previous disinfection any clothing (*i e.*, in such a case his own) which had been exposed to infection. But it is doubtful whether such a prosecution would lie for exposing clothing not the clothing of the sufferer, and at any rate it would be easy for any man to keep his working clothes in a place apart.

It may be observed that amongst those sections of the Public Health Act, 1875, Sects. 120–130, grouped under the common title of "Provisions against Infection," there is one case expressly mentioned in which it is enacted that the local authority may give compensation, viz., when they direct the destruction of any bedding, clothing, or other articles which have been exposed to infection. It may be argued from this, that this is the only case in which under those provisions the legislature contemplated that a case for compensation would arise. *Expressio unius est exclusio alterius.* On the other hand, it may be contended that the provision amounts to a direction that in such cases compensation shall be given, whatever may be done in other cases. But on the whole it may be taken that the law does not give a man who has thrown up his employment in consequence of infectious disease in his family a right to compensation out of the rates.

### Preventing Infectious Disease.

When any member of a family or inmate of a house is suffering from infectious disease, the head of the household is often considerably worried by the formalities and expenses in which he finds himself involved by the law, and which he often considers needless and vexatious as applied to his own case. Sect. 3 of the Infectious Disease (Notification) Act, 1889, enacts that where an inmate of any building used for human habitation is suffering from an infectious disease to which this Act applies, then, unless such building is a hospital in which persons suffering from an infectious disease are received, the following provisions shall have effect, that is to say:—

(*a*) The head of the family to which such patient belongs, and in his default the nearest relatives of the patient present in the building or being in attendance on the patient, and in default of such relatives every person in charge of or in attendance on the patient, and in default of any such person the occupier of the building shall, as soon as he becomes aware that the patient is suffering from an infectious disease to which this Act applies, send notice thereof to the medical officer of health of the district;

(*b*) Every medical practitioner attending on or called in to visit the patient shall forthwith, on becoming aware that the patient is suffering from an infectious disease to which this Act applies, send to the medical officer of health of the district a certificate stating the name of the patient, the situation of the building, and the infectious disease from which, in the opinion of such medical practitioner, the patient is suffering.

Every person required by this section to give a notice or certificate who fails to give the same shall be liable on summary conviction to a fine not exceeding 40s. Provided that if a person is not required to give notice in the first instance, but only in default of some other person, he shall not be liable to any fine if he satisfies the court that he had reasonable cause to suppose that the notice had been duly given.

Sect. 4 enacts that the local authority shall gratuitously supply forms of certificate to any medical practitioner residing or practising in their district who applies for the same, and shall pay to every medical practitioner for each certificate duly sent by him in accordance with this Act a fee of 2s. 6d. if the case occurs in his private practice, and of 1s. if the [illegible]ase occurs in his practice as medical officer of any public [illegible]ly or institution.

Sect. 6 en[illegible]s that in this Act the expression "infectious disease to [illegible]hich this Act applies" means any of the following diseases, namely, small-pox, cholera, diphtheria, membranous croup, erysipelas, scarlatina, scarlet fever, and the fevers known by any of the following names, typhus, typhoid, enteric, relapsing, continued, or puerperal, and includes as respects any particular district any infectious disease to which this Act has been applied by the local authority.

The chief grievance of the respectable British householder so far is that the law tends to over-hasty notification of infections disease, when, perhaps, a day or two's delay would show that the case was not one of scarlet fever at all, but merely nettle rash or something innocuous. And when once the case has been notified there is no *locus pœnitentiæ* for the withdrawal of the notice, and it may be difficult to get the local authority not to insist on disinfection. When a child is taken ill in a seaside lodging or a hired house, for instance, it is a serious matter for the lodging-house keeper or for the owner of the house. It may be desired to remove the patient at once. If so, it must be remembered that a penalty of £5 is incurred for wilful exposure of the sufferer in a public place. The best way of removing the patient is, if possible, in an ambulance provided by the local authority, under Sect. 123 of the Public Health Act, 1875, in which case the expense falls on the local authority. But if there is no such ambulance then it must be remembered that, if a public conveyance is used, the owner or driver thereof must be notified beforehand of the nature of the case, and paid in advance sufficient to cover the expenses of disinfection. In *Mallock* v. *Hunter* (21 Ct. of Sess. 4, sect. 22) a medical practitioner was charged with a contravention of the Public Health Act in that "he being the person in charge of A. B.," a person suffering from enteric fever, being an infectious disorder, "did wilfully expose him while he was suffering as aforesaid from an infectious disorder, by placing him in a cab or hackney carriage, being a public conveyance," and conveying him from his lodgings to a hospital, "without taking proper precautions against spreading the said disorder." It was proved that the accused had taken A. B., while suffering from enteric fever, to a hospital in a cab, being a public conveyance, that the cab was furnished with leather fittings, but there was no evidence as to what other precautions against spreading the disorder were necessary. The magistrate convicted the accused. On appeal, it was held (1) that there was no offence committed, unless the patient was conveyed without proper precaution being taken against endangering the public safety by spreading infection; (2) that the *onus* lay upon the prosecutor to prove what precautions were necessary, and that they had not been used; and (3) that as the prosecutor had failed to discharge this *onus* the conviction must be quashed.

In most cases it is not worth while to remove a patient after the case has been pronounced to be one of infectious disease, except to a hospital. If the patient is removed to a hospital provided by the local authority under Sect. 131 of the Public Health Act, 1875, then the expenses of maintaining him there are (in other than pauper cases) recoverable under Sect. 132 of the act. In places where an isolation hospital has been established under the provisions of the Isolation Hospitals Act, 1893, the patient may be removed to such hospital, and the expenses of the patient, which include the cost of conveying, removing, feeding, providing medicines, disinfecting, etc., are recoverable in non-pauper cases by the hospital committee from the local authority of the area from which the patient is sent, and are, apparently (at least, so far as expenses of maintenance are concerned), recoverable over by the local authority under Sect. 132 of the Public Health Act, 1875.

### Private Nursing of Infectious Disease.

Where the patient is not removed to a hospital, but is privately nursed, it must be borne in mind that in London and in places where that part of the Infectious Disease Prevention Act, 1890, has been adopted, any person who knowingly casts or causes or permits any infectious rubbish (*e.g.* poultices, rags, etc., from the sick-room) to be cast without previous disinfection into any ashpit or other receptacle for refuse matter, thereby renders himself liable to a penalty of £5, and if the offence continues, to a further fine of £2 per day. All combustible odds and ends should be burned and incombustibles should be buried with quick-lime or other disinfectants. The sanitary authority is compelled to give notice to the head of the household of the liability to a fine of any person committing the above offence.

In London all disinfection is practically done by the sanitary authority free of charge. The master or owner of the house and bedding, etc., is bound either to have it done, or allow it to be done, but he need not do it at his own expense, as the expense is ultimately thrown by the law upon the sanitary authority. The sanitary authority are also bound to pay compensation for necessary destruction and unnecessary damage. Outside London the law is not so favourable to the householder. Under section 120 of the Public Health Act, 1875, it is the duty of the sanitary authority to cause the house, etc, to be disinfected, but they may enforce the execution of the work by the householder at his own expense, although they have power instead to take the work and expenses upon themselves. They may also direct the destruction of bedding, etc., and give compensation under Sect. 121, or may disinfect it free of charge under Sect. 122 of the same Act. Where Sect. 5 of the Infectious Disease Prevention Act, 1890, has been adopted, instead of giving the householder a limited time to do the work, the local authority may give him a limited time to choose whether he or they shall do the work.

Throughout the country as well as in London there is a penalty of £20 for letting a house in which there has been a person suffering from infectious disease without having had the house previously disinfected; and the like penalty for making false statements on letting a house in which there has been a person so suffering within six weeks previously. Moreover, in the latter case, one month's imprisonment with hard labour may be given as an alternative punishment.

### Death by Infectious Disease.

Neither in London nor in any place where Sect. 8 of the Infectious Disease Prevention Act, 1890, has been adopted, is it lawful to keep the body in a room used at the same time as a dwelling-place, sleeping-place, or workroom for more than 48 hours without a proper medical certificate. If the death has taken place in a hospital, removal, except for burial, may be prohibited by medical certificate. But if in any case of death, whether in a hospital or not, it is desired to delay the funeral for the arrival of relatives or other reasons, the body should undoubtedly be kept in a mortuary, and public mortuaries are provided in many places either by sanitary authorities under Sect. 141 of the Act of 1875, or by burial boards under Sect. 42 of the Burial Act, 1852.

